# Neuritin Up-regulates Kv4.2 α-Subunit of Potassium Channel Expression and Affects Neuronal Excitability by Regulating the Calcium-Calcineurin-NFATc4 Signaling Pathway*

**DOI:** 10.1074/jbc.M115.708883

**Published:** 2016-06-15

**Authors:** Jin-jing Yao, Qian-Ru Zhao, Dong-Dong Liu, Chi-Wing Chow, Yan-Ai Mei

**Affiliations:** From the ‡ Institutes of Brain Science, School of Life Sciences and State Key Laboratory of Medical Neurobiology, Fudan University, Shanghai 200433, China and; the § Department of Molecular Pharmacology, Albert Einstein College of Medicine, New York, New York 10461

**Keywords:** calcineurin, calcium, neurotrophic factor, potassium channel, transcription factor

## Abstract

Neuritin is an important neurotrophin that regulates neural development, synaptic plasticity, and neuronal survival. Elucidating the downstream molecular signaling is important for potential therapeutic applications of neuritin in neuronal dysfunctions. We previously showed that neuritin up-regulates transient potassium outward current (*I*_A_) subunit Kv4.2 expression and increases *I*_A_ densities, in part by activating the insulin receptor signaling pathway. Molecular mechanisms of neuritin-induced Kv4.2 expression remain elusive. Here, we report that the Ca^2+^/calcineurin (CaN)/nuclear factor of activated T-cells (NFAT) c4 axis is required for neuritin-induced Kv4.2 transcriptional expression and potentiation of *I*_A_ densities in cerebellum granule neurons. We found that neuritin elevates intracellular Ca^2+^ and increases Kv4.2 expression and *I*_A_ densities; this effect was sensitive to CaN inhibition and was eliminated in *Nfatc4*^−/−^ mice but not in *Nfatc2*^−/−^ mice. Stimulation with neuritin significantly increased nuclear accumulation of NFATc4 in cerebellum granule cells and HeLa cells, which expressed IR. Furthermore, NFATc4 was recruited to the Kv4.2 gene promoter loci detected by luciferase reporter and chromatin immunoprecipitation assays. More importantly, data obtained from cortical neurons following adeno-associated virus-mediated overexpression of neuritin indicated that reduced neuronal excitability and increased formation of dendritic spines were abrogated in the *Nfatc4*^−/−^ mice. Together, these data demonstrate an indispensable role for the CaN/NFATc4 signaling pathway in neuritin-regulated neuronal functions.

## Introduction

Neuritin (also known as candidate plasticity gene 15, cpg15) is an important neurotrophin expressed during the development of the nervous system (1). Previous studies showed that neuritin regulates neural development, synaptic plasticity, and neuronal survival (2, 3). Furthermore, neuritin modulates cerebral ischemia, depression, schizophrenia, and cognitive function (4). Thus, further understanding of neuritin molecular signaling pathways is important for potential therapeutic applications of neuritin in neuronal dysfunctions. We recently reported that neuritin activates the MEK-ERK and the Akt mammalian target of rapamycin (mTOR)^4^ pathways in rat cerebellum granule neurons (CGNs) via the insulin receptor (IR) (5). Activation of the IR by neuritin potentiates the expression of the Kv4.2 subunit of transient potassium outward current (*I*_A_) channels at both transcriptional and translational levels. However, the molecular mechanism underlying transcriptional induction of Kv4.2 by neuritin is unknown.

Voltage-gated *I*_A_ channels play a critical role in dampening neuronal excitability and action potential (AP) firing frequency in a wide variety of neurons. In CGNs and hippocampal and cortical neurons, *I*_A_ channels are encoded by pore-forming α-subunits of the Kv4 subfamily (6–8). In particular, the expression of Kv4.2 and its subsequent post-translational regulation are intimately coupled with neuronal excitability (9, 10). Although calcium-activated calcineurin (CaN) increases Kv4.2 transcription in cardiomyocytes (11), it is unclear whether neuronal induction of Kv4.2 expression by neuritin underlies calcium-activated CaN.

Transcription factor nuclear factor of activated T-cells (NFAT), consisting of NFATc1–4, is the key downstream effector of CaN signaling (12, 13). Upon increases in intracellular calcium, NFATcs are dephosphorylated by CaN, thereby promoting translocation of the transcription factor from the cytosol into the nucleus. Once in the nucleus, NFATcs require the cooperative binding of a phosphorylated nuclear partner to initiate transcription (14, 15). Previous studies demonstrated that among the members of NFATc family, NFATc4 plays an important role in neural development, axon growth, and neuronal survival (16–18). In particular, NFATc4 is associated with neurotrophins (*e.g.* brain-derived neurotrophic factor) and depolarization-induced nuclear accumulation of neuronal NFAT (18, 19). Furthermore, in hippocampal neurons NFATc4 modulates expression of γ-aminobutyric acid A receptor subunits (GABR_A2_ and GABR_A4_) by direct transcriptional co-regulation via binding to specific responsive elements in their promoters (20). Thus, induction of Kv4.2 expression and increases in *I*_A_ current density by neuritin should also be possible through the calcium/CaN/NFATc4 pathway.

The goal of this report was to investigate the effect of the calcium/CaN/NFATc4 pathway on neuritin-induced Kv4.2 expression and *I*_A_ current density in mice CGNs. To test our hypothesis, *Nfatc2*^−/−^ and *Nfatc4*^−/−^ double knock-out (DKO) mice were used in this study. In addition, the subsequent effect of neuritin-induced Kv4.2 expression on cortical neuronal excitability was also explored with adeno-associated virus (AAV)-mediated overexpression of neuritin in *Nfatc4*^−/−^ mice. Our study, for the first time, reveals a calcium/CaN/NFATc4 signaling pathway in neuritin-mediated up-regulation of neuronal Kv4.2 expression and examines its role in modulating neuronal excitability in mouse.

## Results

### 

#### 

##### Neuritin Increases I_A_ Densities and Kv4.2 Expression via the Ca^2+^/CaN Pathway

**FIGURE 1.**
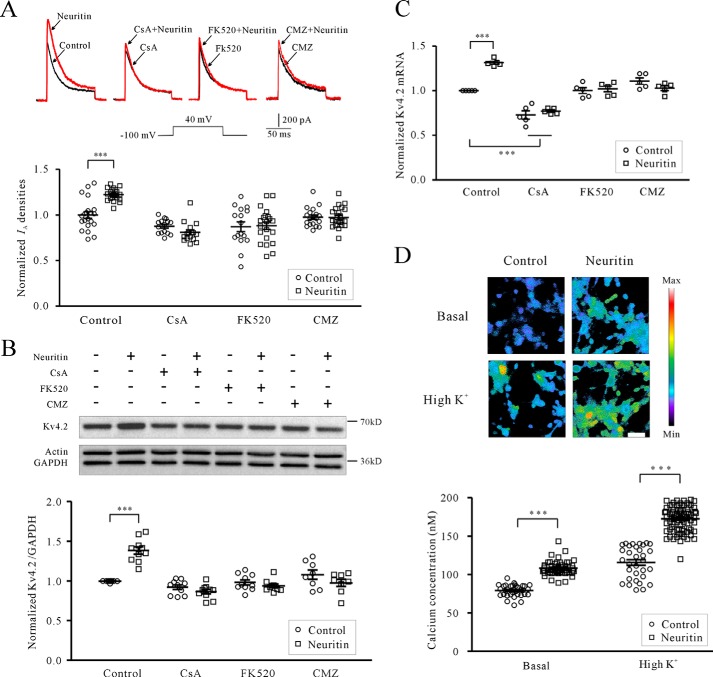
**Neuritin increases *I*_A_ densities and Kv4.2 expression via the Ca^2+^/CaN pathway.** *A*, effects of CaN inhibitor CsA, FK520, or CaM blocker CMZ on neuritin-induced *I*_A_ densities in mouse CGNs. *I*_A_ was evoked by 200 ms of depolarization to +40 mV from a holding potential of −100 mV. CsA, FK520, and CMZ were applied for 2 h prior to initiating treatment with neuritin and then remained in the culture during the entire duration (24 h) of treatment with neuritin (150 ng/ml). *B*, effects of CsA, FK520, or CMZ on neuritin-induced expression of Kv4.2 protein examined by Western blotting. *C*, effects of CsA, FK520, or CMZ on neuritin-induced expression of Kv4.2 mRNA level as measured by quantitative RT-PCR. The data were obtained from five independent experiments. *D*, effect of neuritin on intracellular Ca^2+^ levels was imaged, and statistical analysis was performed before (basal) and after 27 mm KCl depolarization (high K^+^). Changes in the fura-2 fluorescence excitation ratios with increasing [Ca^2+^] are depicted from *purple* to *red. Scale bar*, 30 μm. ***, *p* < 0.001 for two groups connected with a *straight line* by unpaired *t* test or one-way ANOVA followed by Fisher's post hoc test.

To examine whether the Ca^2+^/CaN/NFATc4 pathway plays a role in neuritin-induced *I*_A_ densities, we first employed cyclosporin A (CsA) and FK520 to inhibit CaN, and CsA and FK520 were applied for 2 h prior to initiating treatment with neuritin and then remained in the culture during the entire duration (24 h) of neuritin treatment. *I*_A_ was evoked by 200 ms of depolarization to +40 mV from a holding potential of −100 mV in the presence of 20 mm triethanolamine, which suppresses *I*_K_ and permits better resolution of *I*_A_. The *I*_A_ densities after incubation with 150 ng/ml neuritin for 24 h were significantly increased by 22.42 ± 1.39% (*n* = 43, *p* < 0.01) compared with the control group, whereas in the presence of 5 μm CsA or 0.2 μm FK520 (21), the neuritin-induced increase in *I*_A_ density was reduced to −8.37 ± 2.47% (*n* = 31) and 5.78 ± 5.63% (*n* = 37) for CsA and FK520, respectively (Fig. 1*A*). There was no significant difference compared with the corresponding control.

Inhibition of CaN with CsA and FK520 also abrogated the neuritin-induced increase in Kv4.2 mRNA and protein expression measured by RT-PCR and Western blotting, respectively (Fig. 1, *B* and *C*). CsA at 5 μm or FK520 at 0.2 μm significantly reduced the induction of Kv4.2 mRNA from 31.54 ± 1.71% (*n* = 5) for neuritin alone to 5.94 ± 3.47% (*n* = 5) and 1.87 ± 3.28% (*n* = 5) for CsA and FK520, respectively (Fig. 1*C*). The induction in Kv4.2 protein was also significantly reduced from 38.40 ± 3.30% (*n* = 10) for neuritin alone to −6.07 ± 2.84% (*n* = 10) using CsA and to −4.45 ± 2.79% (*n* = 10) using FK520 (Fig. 1*B*). These data indicate that CaN contributes to neuritin induction of Kv4.2 expression and its subsequent potentiation of *I*_A_ densities. By using the one-way ANOVA analysis, the data presented in Fig. 1*C* showed there was significant difference between control and CsA, but there was no significant difference between CsA and CsA with neuritin. Moreover, there was no significant difference between control and FK520. These phenomena suggested that CsA may affect the transcriptional expression of Kv4.2 under basal conditions by no calcineurin-dependent pathway.

Increases in intracellular Ca^2+^ concentration and CaM activity is a prerequisite for CaN activation. Thus, we examined the intracellular Ca^2+^ ([Ca^2+^]*_i_*) level in CGNs following neuritin stimulation using the Ca^2+^-sensitive fluorescent dye fura-2 acetoxymethyl (AM). *In vitro* calcium imaging showed that neuritin increased the [Ca^2+^]*_i_* level in mouse CGNs at both basal and high K^+^ conditions (Fig. 1*D*). Under basal conditions, neuritin induced an increase in the Ca^2+^ concentration from 79.19 ± 1.32 nm (*n* = 34) to 108.40 ± 1.23 nm (*n* = 59, *p* < 0.001). Using a high K^+^ solution (27 mm KCl) to depolarize neurons and activate voltage-gated Ca^2+^ channels caused a rapid elevation in [Ca^2+^]*_i_*. Following incubation of CGNs with neuritin for 15 min, depolarization with high K^+^ induced an increase in the Ca^2+^ concentration that was enhanced from 115.70 ± 3.72 nm (*n* = 32) to 172.60 ± 1.85 nm (*n* = 71, *p* < 0.001). Similar to CaN inhibition, blocking CaM function using calmidazolium chloride (CMZ) also abrogated the induction of *I*_A_ densities, Kv4.2 mRNA, and protein expression by neuritin (Fig. 1, *A–C*). *I*_A_ densities, Kv4.2 mRNA levels, and protein expression induced by neuritin were reduced from 22.42 ± 1.39% (*n* = 43), 31.54 ± 1.71% (*n* = 5) and 38.40 ± 3.30% (*n* = 8), respectively, to 0.32 ± 2.34% (*n* = 42), −7.18 ± 3.19% (*n* = 5), and −9.59 ± 5.27% (*n* = 8), respectively, in the presence of CMZ. Together, these data suggest that neuritin stimulation increases intracellular Ca^2+^ levels in CGNs, which activate CaN for the induction of Kv4.2 transcription and *I*_A_ densities.

##### Neuritin-induced Kv4.2 Expression and I_A_ Densities Require NFATc4

**FIGURE 2.**
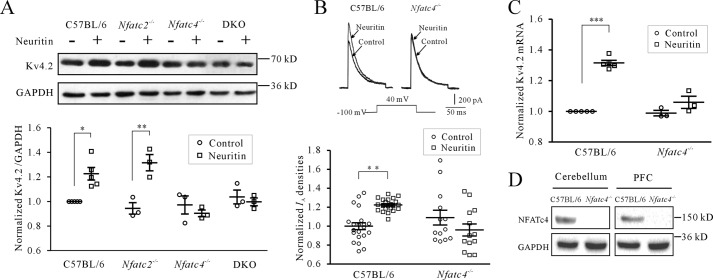
**Neuritin-induced Kv4.2 expression and *I*_A_ densities require NFATc4.** *A*, effect of neuritin on Kv4.2 protein expression in CGNs isolated from wild type mice (C57BL/6), *Nfatc2*^−/−^, *Nfatc4*^−/−^, and the combined *Nfatc2*^−/−^ and *Nfatc4*^−/−^ DKO mice measured by Western blotting. *B*, induction of *I*_A_ densities recording from C57BL/6 and *Nfatc4*^−/−^ mouse CGNs after neuritin stimulation. *C*, induction of Kv4.2 mRNA level measured by quantitative RT-PCR from C57BL/6 and *Nfatc4*^−/−^ mouse CGNs after neuritin stimulation. The data were obtained from three independent experiments. *D*, representative sample showing Western blotting-detected expression of NFATc4 protein in mouse cerebellum and PFC from C57BL/6 and *Nfatc4*^−/−^ mice. *, *p* < 0.05; **, *p* < 0.01; and ***, *p* < 0.001 for two groups connected with a *straight line* by one-way ANOVA followed by Fisher's post hoc test.

Members of the transcription factor NFATc family, consisting of NFATc1–4, are the key downstream effectors in the Ca^2+^/CaN pathway (22). Because it is the NFAT isoforms NFATc1/c2 and NFATc4 that regulate the transcription of M-type (Kv7) K^+^ channels, as well as GABR_A2_ and GABR_A4_ subunit expression in neurons (20, 23), we first examined the effect of neuritin on Kv4.2 expression in CGNs isolated from *Nfatc2*^−/−^, *Nfatc4*^−/−^, and combined *Nfatc2*^−/−^ and *Nfatc4*^−/−^ DKO mice. We observed a similar induction of Kv4.2 by neuritin in *Nfatc2*^−/−^ and wild type CGNs. The expression of Kv4.2 protein stimulated with neuritin in *Nfatc2*^−/−^ and wild type CGNs was significantly increased by 39.40 ± 5.70% (*n* = 3, *p* < 0.05) and 22.60 ± 5.12% (*n* = 5, *p* < 0.05), respectively, compared with the control group (Fig. 2*A*). By contrast, induction of Kv4.2 by neuritin was abolished in CGNs isolated from *Nfatc4*^−/−^ or DKO mice (Fig. 2*A*). The expression of Kv4.2 protein in CGNs isolated from *Nfatc4*^−/−^ or DKO mice was changed by only −7.05 ± 5.56% (*n* = 3) and −3.83 ± 4.56% (*n* = 3), respectively, with no significant difference compared with control. Based on these data, we thus exploited *Nfatc4*^−/−^ mice to directly examine whether NFATc4 was required for the neuritin-induced increase in Kv4.2 expression and *I*_A_ densities.

Correspondingly, *I*_A_ densities recorded by patch clamp and Kv4.2 mRNA levels measured by quantitative RT-PCR were not induced by neuritin in *Nfatc4*^−/−^ mouse CGNs (Fig. 2, *B* and *C*). In *Nfatc4*^−/−^ mice, CGN *I*_A_ densities and Kv4.2 mRNA levels after treatment with neuritin were changed minimally by −11.97 ± 7.13% (*n* = 26) and 7.14 ± 3.07% (*n* = 3), respectively. These data indicate that NFATc4 is required for the induction of Kv4.2 expression and increases in *I*_A_ densities by neuritin. We simultaneously confirmed the lack of NFATc4 expression in the cerebellum and PFC of *Nfatc4*^−/−^ mice by Western blotting. The results indicated that neither cerebellum nor PFC neurons in *Nfatc4*^−/−^ mice expressed NFATc4 (Fig. 2*D*).

##### Neuritin Induces Dephosphorylation and Nuclear Accumulation of NFATc4 to Control Kv4.2 Transcription

**FIGURE 3.**
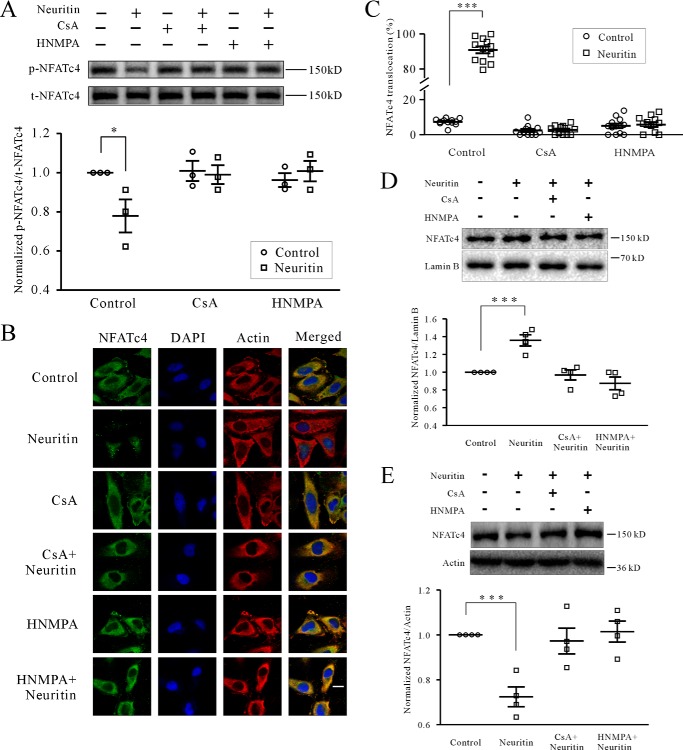
**Neuritin induces dephosphorylation and nuclear accumulation of NFATc4.** *A*, the levels of NFATc4 Ser^168^ and Ser^170^ phosphorylation (*p-NFATc4*) after neuritin treatment for 20 min in mice CGNs were examined by Western blotting. *B* and *C*, representative recording sample and statistical analysis showing effect of neuritin on nuclear accumulation of NFATc4 was examined by confocal microscopy in HeLa cells, which expressed IR activated by neuritin. The effects of CaN inhibitor CsA and IR inhibitor HNMPA are also shown. The percentage of nuclear NFATc4 in 100 cells was counted and presented. *D*, effect of neuritin on nuclear NFATc4 protein in mice CGNs by Western blotting analysis. *E*, effect of neuritin on cytoplasmic NFATc4 protein in mice CGNs by Western blotting analysis. *, *p* < 0.05; and ***, *p* < 0.001 for two groups connected with a straight line by one-way ANOVA followed by Fisher's post hoc test.

CaN dephosphorylates key Ser residues in the NFAT homology domain of NFATc4, including Ser^168^ and Ser^170^, and facilitates nuclear accumulation of NFATc4 (21). Thus, we examined the phosphorylation status and nuclear localization of NFATc4 upon neuritin stimulation in mice CGNs. After pretreatment of CGNs with neuritin for 20 min, phosphorylation on Ser^168^ and Ser^170^ of NFATc4 was significantly reduced by 22.12 ± 8.42% (*n* = 3, *p* < 0.05) compared with control cells (Fig. 3*A*). Meanwhile, either CaN inhibition or blocking neuritin signaling via the IR inhibitor hydroxy-2-naphthalenylmethyl phosphonic acid (HNMPA) (24, 25) abolished dephosphorylation of NFATc4 induced by neuritin. In the presence of 5 μm CsA or 100 μm HNMPA, phosphorylation on Ser^168^ and Ser^170^ of NFATc4 compared with controls was not significantly reduced by only 1.88 ± 5.00% (*n* = 3) and −4.80 ± 4.46% (*n* = 3), respectively (Fig. 3*A*). These data indicate that activation of CaN by neuritin promotes dephosphorylation of NFATc4.

Next, we examined the localization of NFATc4 in HeLa cells by confocal microscopy because they express IR activated by neuritin (5) and have a larger cell body. As expected, NFATc4 was mainly detected in the cytoplasm at the resting state. Stimulation with neuritin for 20 min significantly increased nuclear accumulation of NFATc4, which was blocked by CsA and HNMPA (Fig. 3*B*). Static analysis indicated that the percentage of NFATc4 translocation in HeLa cells was significantly increased by 1125.99 ± 1.43% (*n* = 12, *p* < 0.001) following neuritin stimulation (Fig. 3*C*). In the presence of 5 μm CsA or 100 μm HNMPA, the percentage of NFATc4 nuclear translocation stimulated by neuritin was reduced to 13.07 ± 11.24% (*n* = 8) and 13.47 ± 16.62% (*n* = 9), respectively, which were not significantly different from the percentage of translocation when neuritin was applied alone.

Nuclear and cytoplasmic protein extraction and Western blotting analysis in mice CGNs also indicated that nuclear NFATc4 was increased significantly by 35.89 ± 6.33% (*n* = 4, *p* < 0.01), and cytoplasmic NFATc4 was decreased by 27.59 ± 4.40% (*n* = 4, *p* < 0.01) following neuritin stimulation, whereas this effect was blocked by CsA and HNMPA (Fig. 3, *D* and *E*). In the presence of 5 μm CsA or 100 μm HNMPA, the percentage of NFATc4 translocation to nuclear stimulated by neuritin was reduced to −3.08 ± 5.68% (*n* = 4) or −11.8 ± 6.86% (*n* = 4), respectively. On the other hand, neuritin-induced decrease of cytoplasmid NFATc4 was reduced to 2.74 ± 5.73% (*n* = 4) or −1.43 ± 4.68% (*n* = 4), respectively, and this is not significantly different from the cells treated with neuritin alone. Together, these data indicate that neuritin activates and translocates NFATc4 into neuronal nuclei.

**FIGURE 4.**
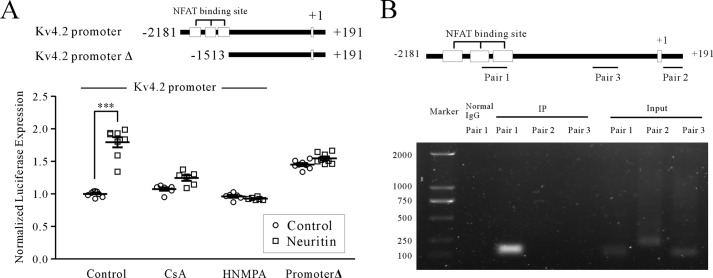
**Effect of neuritin on the Kv4.2 gene promoter.** *A*, effect of neuritin on the Kv4.2 gene promoter (−2181 to +191 bp) was determined by luciferase reporter assays. The effect of deleting NFAT binding sites to −1513 bp (promoter Δ) is shown. *B*, recruitment of NFATc4 to the Kv4.2 gene promoter was examined by chromatin immunoprecipitations. Primers for PCR amplification (pairs 1, 2, and 3) are illustrated. ***, *p* < 0.001 for two groups connected with a straight line by one-way ANOVA followed by Fisher's post hoc test.

A study showed that NFAT plays an important role in electroexcitability in the heart by regulating Kv4.2 transcription in cardiomyocytes (11). Indeed, specific upstream NFAT enhancers were identified in the Kv4.2 gene promoter. Thus, we examined the effect of neuritin on the Kv4.2 gene promoter using luciferase reporter assays. Administration of neuritin significantly increased luciferase expression driven by the mouse Kv4.2 promoter by 79.45 ± 5.60% (*n* = 8; *p* < 0.001; Fig. 4*A*). Induction of Kv4.2 promoter activity by neuritin, however, was abrogated by 5 μm CsA or 100 μm HNMPA, reducing expression to 15.90 ± 3.61% (*n* = 6) and −3.57 ± 1.61% (*n* = 6), respectively, which were not significantly different from corresponding controls. In addition, deletional mutagenesis of the NFAT enhancer to −1513 bp on the Kv4.2 promoter (promoter Δ) also abolished neuritin-induced Kv4.2 promoter activity (Fig. 4*A*). These data indicate that neuritin-induced Kv4.2 transcription is NFAT-dependent.

We further ascertained recruitment of NFATc4 to the Kv4.2 gene loci by ChIP assays (Fig. 4*B*). Kv4.2 promoter DNA encoding the NFAT enhancers was detected in NFATc4 precipitates, whereas neither downstream NFAT enhancers nor the 5′-untranslated region of the Kv4.2 gene was detected. Together, these data confirm that neuritin regulates Kv4.2 gene transcription via the CaN/NFATc4 pathway.

##### Requirement of NFATc4 in Neuritin-mediated Neuronal Excitability and Formation of Dendritic Spines

The Kv4 subfamily encodes critical regulatory components of voltage-gated *I*_A_ channels, which dampen neuronal excitability and action potential firing frequency (26). Induction of Kv4.2 expression and *I*_A_ densities in rodent CGNs indicate that neuritin may alter neuronal excitability *in vivo*. Given that neuritin activates the CaN/NFATc4 pathway, we hypothesized that *Nfatc4*^−/−^ mice might exhibit defects in neuronal excitability upon neuritin stimulation.

**FIGURE 5.**
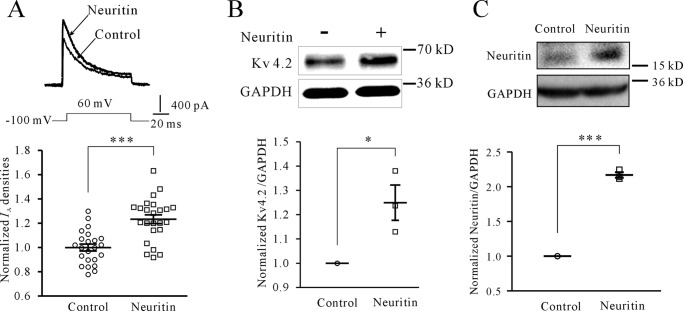
**Neuritin has the same effect on *I*_A_ densities and Kv4.2 protein expression in cortical neuron.** *A*, induction of *I*_A_ densities by current recording in C57BL/6 mouse cortical neurons upon neuritin stimulation. The *upper panel* shows a representative sample, and statistical analysis is shown in the *lower panel*. ***, *p* < 0.001 compared with corresponding control by unpaired *t* test. *B*, Kv4.2 protein expression by Western blotting in C57BL/6 mouse cortical neurons upon neuritin stimulation. *, *p* < 0.05 compared with value 1 as the hypothetical mean by one-sample *t* test. The *upper panel* shows a representative sample, and statistical analysis is shown in the *lower panel. C*, expression level of neuritin after AAV-control and AAV-neuritin virus injected bilaterally into the PFC for 2 weeks was determined by Western blotting. ***, *p* < 0.001 compared with value 1 as the hypothetical mean by one-sample *t* test.

To test this hypothesis, we first ascertained whether neuritin induces similar potentiation of *I*_A_ densities and induction of Kv4.2 expression in cortical neurons as in CGNs. Both *I*_A_ densities and Kv4.2 protein expression in cortical neurons were significantly increased by 23.34 ± 3.28% (*n* = 47, *p* < 0.05) and 24.94 ± 7.27% (*n* = 3, *p* < 0.05), respectively, after incubation of cortical neurons with 150 ng/ml neuritin for 24 h (Fig. 5, *A* and *B*). Next, we employed AAV9-mediated transduction to express exogenous neuritin following injection into the prefrontal cortex (PFC) (0.2 μl/mouse). Western blotting analysis indicated that expression of neuritin was increased by 117 ± 3.89% (*n* = 3, *p* < 0.05) after viral expression of exogenous neuritin in the PFC for 2 weeks (Fig. 5*C*).

**FIGURE 6.**
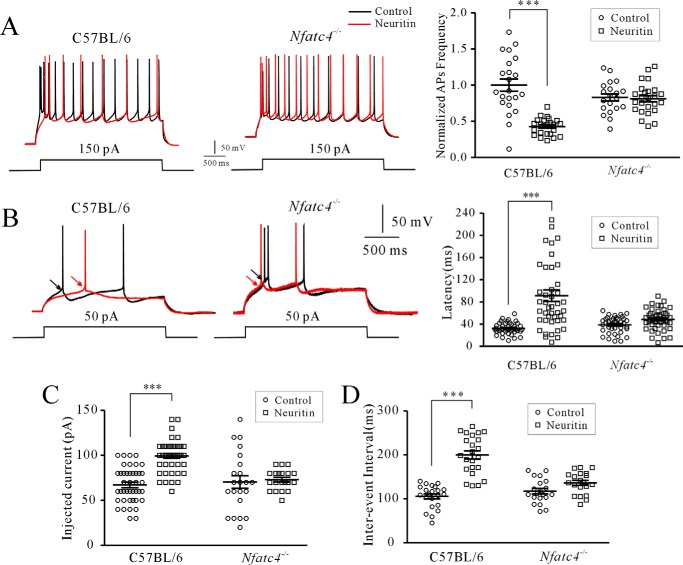
**Requirement of NFATc4 in neuritin-mediated neuronal excitability in PFC neurons of mice.** *A*, effect of AAV-mediated neuritin expression on AP firing frequency in C57BL/6 and *Nfatc4*^−/−^ mouse cortical neurons. *B*, effect of AAV-mediated neuritin expression on the latency of the first AP in C57BL/6 and *Nfatc4*^−/−^ mouse cortical neurons. *C* and *D*, effect of AAV-mediated neuritin expression on injected current to evoke the first AP and interevent interval in C57BL/6 and *Nfatc4*^−/−^ mouse cortical neurons. ***, *p* < 0.01 for two groups connected with a straight line by one-way ANOVA followed by Fisher's post hoc test.

We then measured the AP from layer II–III of pyramidal neurons in mouse PFC. Overexpression of neuritin significantly decreased AP firing frequency by 57.17 ± 6.22% (*n* = 45, *p* < 0.001; Fig. 6*A*), whereas the latency of the first AP, the injected current to evoke the first AP, and the interevent interval increased significantly by 182.38 ± 22.62% (*n* = 86, *p* < 0.01), 47.94 ± 4.24% (*n* = 87, *p* < 0.05), and 89.34 ± 5.24% (*n* = 45, *p* < 0.001), respectively, in native control mice (Fig. 6, *B–D*). Changes in neuronal excitability following neuritin overexpression, however, were abrogated in the *Nfatc4*^−/−^ mice. The AP firing frequency, the latency of the first AP, the injected current to evoke the first AP, and the interevent interval did not differ significantly in *Nfatc4*^−/−^ mice with and without neuritin overexpression (Fig. 6, *A–D*). These data indicate that neuritin modulation of neuronal excitability in the mouse PFC is NFATc4-dependent.

**FIGURE 7.**
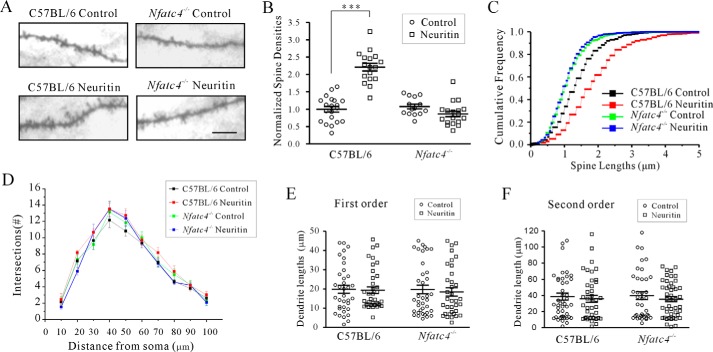
**Requirement of NFATc4 in neuritin-mediated formation of dendritic spines in PFC neurons.** *A* and *B*, representative images and statistical analyses of the effect of neuritin on PFC neuron spine density in C57BL/6 and *Nfatc4*^−/−^mice. Identification of neurons was performed using Golgi staining. *C*, effect of neuritin on spine length of PFC neurons in C57BL/6 or *Nfatc4*^−/−^mice. Cumulative frequency distributions of spine length were analyzed using a one-way Kruskal-Wallis test followed by Kolmogorov-Smirnov test. The median and quartiles of the control group and neuritin-treated group were compared. *D*, Sholl analysis of the branch intersection numbers. *E* and *F*, effect of neuritin on first and second order dendrite length of PFC neurons in C57BL/6 or *Nfatc4*^−/−^ mice. ***, *p* < 0.001 compared with corresponding control by one-way ANOVA followed by Fisher's post hoc test.

We also examined whether the increased formation of dendritic spines by neuritin reported previously (27) requires NFATc4 using Golgi staining. Notably, Kv4.2 channels are mainly localized at dendritic spines, and dendritic spine densities are associated with synaptic excitability (28, 29). Our results confirmed that spine density was significantly increased by 120.84 ± 9.73% (*n* = 37, *p* < 0.001) in native control mice upon neuritin expression (Fig. 7, *A* and *B*). Cumulative frequency distributions also showed significantly increased spine lengths after neuritin treatment (*n* = 37, *p* < 0.001) analyzed using a one-way Kruskal-Wallis test. The median and quartiles in the control group and the neuritin-treated group were 0.992 (0.784, 0.998) and 0.957 (0.559, 0.996), respectively (Fig. 7*C*). However, the effect of neuritin overexpression on the increase in spine density and its length was abrogated in the *Nfatc4*^−/−^ mice (Fig. 7, *A–C*), in which the median and quartiles of *Nfatc4*^−/−^ mice treated with neuritin were 1.00 (0.939, 1.00). In addition, the effect of neuritin on the number of neurite branches and lengths of the first order and second order apical dendrites in native control and *Nfatc4*^−/−^ mice was analyzed with Sholl and two-way ANOVA analysis. Neuritin increased the number of neurite branches neither in the control mice nor in *Nfatc4*^−/−^ mice (Fig. 7*D*), and the dendrite lengths of the first order and second order were not significantly different between the four groups (Fig. 7, *E* and *F*). Taken together, these data indicate that neuritin signals through the Ca^2+^/CaN/NFATc4 pathway and modulates neuronal excitability and formation of dendritic spines *in vivo*.

## Discussion

Although neuritin, as a new neurotrophic factor, was known to play multiple roles in the process of neural development and synaptic plasticity, little is known about the receptor binding neuritin and its downstream signaling effectors. Our previous study indicated that neuritin specifically increased the densities of *I*_A_ in rat CGNs by increasing mRNA and protein expression of Kv4.2 via the IR pathway (5). In this report, we further revealed the mechanism by which the Ca^2+^/CaM/CaN/NFATc4-dependent pathway mediated transcriptional up-regulation of Kv4.2 expression induced by neuritin.

Elevations in [Ca^2+^]*_i_* led to activation of the Ca^2+^/CaM-dependent protein phosphatase CaN, which then dephosphorylates NFAT (12, 13). Although CaN increases Kv4.2 transcription in cardiomyocytes (11), whether neuronal induction of Kv4.2 expression by neuritin also underlies calcium-activated CaN remains elusive. Here, we show that an increase in intracellular Ca^2+^ levels by neuritin potentiates the transcription of Kv4.2 via the Ca^2+^/CaN-dependent pathway. Moreover, neuritin not only elicited a rise in basal [Ca^2+^]*_i_* but also increased high K^+^-induced rises in [Ca^2+^]*_i_*. Elevated KCl increases [Ca^2+^]*_i_* levels in mouse CGNs via L-type voltage-gated Ca^2+^ channels (30), and the increase in basal [Ca^2+^]*_i_* was known to depend on calcium release from intracellular calcium stores (31). Previous studies in dorsal root ganglion sensory neurons indicated that CaN/NFAT signals can be triggered by multiple Ca^2+^ mechanisms, including the influx of Ca^2+^ from voltage-gated Ca^2+^ channels opened by high K^+^ stimulation, trains of action potentials or opening of TRPV channels to depolarization (32), and inositol 1,4,5-trisphosphate-mediated Ca^2+^ release (33). We thus surmised that either Ca^2+^ influx through Ca^2+^ channels or release from intracellular stores may be involved because neuritin is triggered downstream of the CaM/CaN/NFATc4 pathway. The precise mechanism for the elevation of intracellular Ca^2+^ concentration by neuritin, however, needs further exploration.

Among the different NFAT isoforms, NFATc4 was previously implicated in neuronal development and function, including axonogenesis and survival, neurotrophin signaling, and memory formation in mice (18, 21, 34, 35). Although our study only excluded the role of NFATc2, which was previously reported to be involved in the transcriptional regulation of K^+^ channels in neurons (23), and did not detect an effect of NFATc1 or NFATc3 on neuritin-induced Kv4.2 expression, our data obtained from *Nfatc4*^−/−^ mice nonetheless suggested that the NFATc4 isoform is essential for neuritin signaling in the induction of neuronal Kv4.2 expression. This result is in line with the study of Ding *et al.* (36), in which numerous NFATc4 neuronal targets were identified and NFATc4 was implicated as an important direct regulator of gene expression in CGNs. However, despite similar transcriptional regulation of Kv4.2, NFATc associated with the increase of Kv4.2 promoter activity appears to act differently. Here, the neuritin/Ca^2+^/CaN pathway translocated NFATc4 subtypes to increase Kv4.2 promoter activity in CGNs, but Kv4.2 transcription in neonatal rat ventricular myocytes was associated with NFATc3 activation when CaN was overexpressed in those cells (11). This possibly occurred because distinct NFATc subtypes are activated in different cell types. However, in adult mouse ventricular myocytes, activation of CaN/NFATc3 after myocardial infarction or chronic β-adrenergic stimulation reduced Kv4.2 expression (37, 38). These contrasting effects of NFATc3 on Kv4.2 expression in neonatal rat ventricular myocytes and adult mouse ventricular myocytes suggested that even in the same heart organ, the transcriptional function of NFATc3 may vary among animal species or with developmental status. Whether this phenomenon also occurs in NFATc4 and the nervous system will require further study.

*I*_A_ channels, which are regulated by the Kv4 family, were previously implicated in the control of neuron excitability (39). Recent investigation has indicated that the *I*_A_ channels of mature cortical neurons were encoded by Kv4.2, Kv4.3, and Kv1.4, which differentially regulate intrinsic excitability (39). Up-regulation of Kv4.2 expression and *I*_A_ density by neuritin was also observed in cultured cortical neurons of mice, suggesting that the effect of neuritin on *I*_A_ channels is not specific to CGNs and may be universal in brain neurons, at least in cortical neurons. Here, we utilized cortical neurons in acute slices, which are more likely to elicit action potentials by experimental injection of current. NFATc4 staining has been previously observed in cortical neurons (40), showing a role for CaN/NFATc4-dependent up-regulation of *I*_A_ channels in modulating neuronal excitability in mice. In our study, overexpression of neuritin by AAV-mediated gene infection decreased AP firing frequency in cortical neuron, but this effect could not be reproduced in cortical neurons from NFAT4^−/−^ mice. Our findings thus demonstrate a role for NFATc4 in the regulation of neuronal excitability and provide insights into the molecular and transcription-dependent regulation of neural excitability in cortical neurons of mice. Because the latency of the first AP increased in NFAT4c-deficient mice, it is likely that NFATc4 also affects protein expression of another ion channel, which is associated with latency in native control mice.

Neuritin was first discovered because of its role promoting dendrite/synapse formation (2, 3). In a chronic unpredictable stress model, re-expression of neuritin was sufficient to reduce depression symptoms, in part by increasing dendritic spine density (41). We recently reported that overexpression of hippocampal neuritin using an AAV vector significantly increased the neuritin level and dendritic spine density and reversed deficits in murine novel object associative recognition memory caused by exposure to extremely low frequency (50 Hz) electromagnetic fields (42). The current study showed that neuritin-potentiated spine density and increased dendritic length were regulated by NFATc4. Thus, the neuritin/Ca^2+^/CaN/NFATc4 signaling pathway may potentially act throughout the nervous system to limit overexcitability associated with disease states such as epilepsy and mental dysfunctions and may be a potential therapeutic target for disorders associated with deficits in recognition memory. Further studies will be required to examine this hypothesis.

**FIGURE 8.**
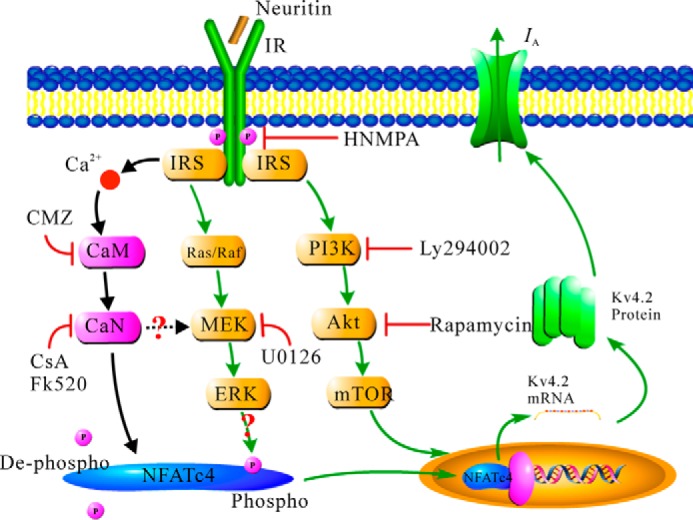
**Schematic illustration depicting Ca^2+^/CaN-dependent NFATc4 activity on the modulation of neuritin-induced Kv4.2 expression and the subsequent increase in *I*_A_ channel density.**

We previously reported that neuritin-up-regulated Kv4.2 expression was associated with the activation of both MEK-ERK and Akt-mTOR pathways in rat CGNs via the IR (5). A recent observation by Ulrich *et al.* (19) demonstrated that inhibition of p38 or mTOR kinases had no significant effect on translocation of NFATc4 in dorsal root ganglion neurons, which may be due to the fact that the Akt-mTOR pathway is more specific to translational processes during protein synthesis (43). This study further suggested that there was cross-talk between CaN and ERK in the CaN-NFATc4 pathway even though it was the Akt-mTOR pathway that was involved in neuritin-induced Kv4.2 expression (5). Our data are consistent with the observation of Ulrich *et al.* (19) that inhibition of p38 or mTOR kinases had no significant effect on translocation of NFATc4 in dorsal root ganglion neurons. Studies by Yang *et al.* (44) and Chandrasekar *et al.* (45) have shown that activation of ERK resulted in phosphorylation of Ser^676^ in NFATc4, which led to greater DNA binding of NFATc4 and provides an additional mechanism for the modulation of NFATc4 transcriptional activity. Thus, it is possible that neuritin activates the CaM-CaN pathway, which dephosphorylates NFATc4 at Ser^168^ and Ser^170^ and facilitates NFATc4 translocation into the nucleus. Meanwhile, neuritin also activates the Ras/Raf/ERK pathway, which phosphorylates NFATc4 at Ser^676^ and increases the DNA binding of NFATc4. These two signal pathways may thus work together to provide optimal conditions for NFATc4 to modulate transcription of the Kv4.2 gene. Further research will be needed to establish the existence of cross-talk between CaN and ERK. Fig. 8 is proposed as a model depicting the mechanisms likely involved in the transcriptional modulation of Kv4.2 expression by the CaN/NFATc4 signaling pathway in cerebellar GCs.

In conclusion, we demonstrate here that neuritin activates the CaM-CaN-NFATc4 pathway to increase the transcription of Kv4.2 and *I*_A_ densities in mouse CGNs. This follows our previous finding that neuritin up-regulated Kv4.2 by activation of the IR and suggests a mechanism underlying the connection between Ca^2+^/CaN/NFATc4-dependent regulation of K^+^ channel transcription and neuronal excitability induced by neuritin. In addition, these studies indicated for the first time that the increase in spine density and dendritic length by neuritin in cortical neurons was also associated with the Ca^2+^/CaN/NFATc4 signaling pathway. Further research will be required to demonstrate the relationship between neuritin-induced up-regulation of Kv4.2 expression and morphological changes in neurons.

## Experimental Procedures

### 

#### 

##### Chemicals

Recombinant human neuritin was purchased from Pepro Tech (Rocky Hill, NJ). Poly-l-lysine, triethanolamine, tetrodotoxin, CsA, ascomycin (FK520), and CMZ were obtained from Sigma. FBS, DMEM, and antibiotic-antimycotic solution were purchased from Invitrogen, Thermo Fisher Scientific. HNMPA was purchased from Santa Cruz Biotechnology (Dallas, TX).

##### Cell Culture

CGNs were derived from cerebella of 7-day-old C57BL/6 mouse pups (SLC Co., Ltd., Shanghai, China) as described previously (46). Isolated cells were seeded in 35-mm Petri dishes (Shenyou, Shanghai, China) coated with poly-l-lysine (10 μg/ml) or on coverslips in Petri dishes coated with poly-l-lysine (50 μg/ml) at a density of 10^5^ cells/cm^2^. Cultured cells were incubated at 37 °C under 5% CO_2_ in DMEM supplemented with 10% FBS, insulin (5 μg/ml), KCl (5 mm), and 1% antibiotic-antimycotic solution. Cytosine β-d-arabinofuranoside (5 μm) was added to the culture medium 24 h after seeding to inhibit the proliferation of non-neuronal cells. The cells were used for experiments after 2–3 days in culture unless otherwise indicated. HeLa cells were maintained as a monolayer in high glucose DMEM, supplemented with 10% FBS and 1% antibiotic-antimycotic solution.

##### Patch Clamp Recordings

Whole cell currents of CGNs were recorded at room temperature using a conventional patch clamping technique (5). Prior to *I*_A_ current recording, the culture medium was replaced with a bath solution containing 125 mm NaCl, 2.5 mm KCl, 10 mm HEPES (pH 7.4), 1 mm MgCl_2_, 0.001 mm tetrodotoxin, 20 mm triethanolamine, and 10 mm glucose. Soft glass recording pipettes were filled with an internal solution containing 135 mm K^+^-gluconate, 10 mm KCl, 10 mm HEPES (pH 7.3), 1 mm CaCl_2_, 1 mm MgCl_2_, 10 mm EGTA, 1 mm ATP, and 0.1 mm GTP. The pipette resistance was 4–6 MΩ after filling with internal solution.

Coronal brain slices (200 μm) containing PFC were prepared using standard methods (47). The slices were transferred to a submerged recovery chamber containing oxygenated (95% O_2_ and 5% CO_2_) artificial cerebral spinal fluid containing 125.0 mm NaCl, l 2.5 mm KC, 2.5 mm CaCl_2_, 1.5 mm MgSO_4_, 26.2 mm NaHCO_3_, 1.0 mm NaH_2_PO_4_, and 11.0 mm glucose at room temperature for at least 1 h. Layers II/III of medial pyramidal neurons in the PFC were visualized using infrared differential interference contrast video microscopy with a 40× water immersion objective, and images were captured with a charge-coupled device camera. Patch pipettes (5–8 MΩ) were filled with 150.0 mm K^+^-gluconate, 0.4 mm EGTA, 8.0 mm NaCl, 2.0 mm ATP, 0.1 mm GTP-NH_3_, and 10.0 mm HEPES (pH 7.3–7.4) at 280 ± 5 mOsm for the recording.

##### Western Blotting Analysis

Mice were anesthetized with pentobarbital sodium (50 mg/kg) and decapitated. The brains were removed immediately. Coronal sections (200 μm) were made at the level of the prefrontal cortex by vibratome (DTK-1000; DSK, Kyoto, Japan). The prefrontal region was then subdissected with a dissecting microscope. After subdissection, the prefrontal tissues were moved into ice-cold Eppendorf tubes and homogenized with a grinding rod on ice.

The homogenized tissue or cultured cells were lysed in HEPES-Nonidet P-40 lysis buffer (20 mm HEPES, 150 mm NaCl, 0.5% Nonidet P-40, 10% glycerol, 2 mm EDTA, 100 μm Na_3_VO_4_, 50 mm NaF, pH 7.5, and 1% protease inhibitor mixture) on ice for 30 min. After centrifugation at 13,800 × *g* for 15 min, the supernatant was mixed with 2× SDS loading buffer and boiled for 5 min. A nuclear and cytoplasmic protein extraction kit (Beyotime, Haimen, China) was used for nuclear and cytoplasmic protein extraction following the instructions of the kit as previously described (48). Before loading, the concentration of total protein in each group was determined using a microplate photospectrometer (multi SKAN MK3; Thermo Fisher Scientific). Proteins were loaded based on the concentrations of total protein to assure equal quantities per lane. Proteins were separated on a 10% SDS-polyacrylamide gel and transferred to PVDF membranes (Millipore, Billerica, MA). The membranes were blocked with 10% nonfat milk and incubated at 4 °C overnight with one of the following antibodies: mouse monoclonal antibody against Kv4.2 (1:2000, catalog no. 75-016; University of California, Davis, CA), rabbit polyclonal antibody against phosphorylated NFATc4 (49, 50), or total NFATc4 (1:1000, catalog nos. sc-32630 and sc-13036, respectively; Santa Cruz Biotechnology), goat polyclonal antibody against neuritin (1:500, catalog no. AF283; R&D Systems, Minneapolis, MN), goat polyclonal antibody against Lamin B (1:1000, catalog no. sc-6217; Santa Cruz Biotechnology), or mouse monoclonal antibody against GAPDH (1:10,000, catalog no. KC-5G4; Kang Chen Bio-Tech, Shanghai, China). After washing in TBS with 0.3% Tween three times for 45 min, the membranes were incubated with HRP-conjugated anti-mouse or anti-rabbit IgG (1:10,000, catalog nos. KC-MM-035 and KC-RB-035, respectively; Kang Chen Bio-Tech), or anti-goat IgG (1:1000, catalog no. A0181; Beyotime) for 2 h at room temperature. Chemiluminescent signals were generated using a Super Signal West Pico trial kit (Pierce Protein Biology, Thermo Fisher Scientific) and detected using the ChemiDoc XRS System (Bio-Rad). Image Lab software (Bio-Rad) was used for background subtraction and for quantification of immunoblotting data. The quantitative linearity of all Western blots was confirmed in separate experiments.

##### Quantitative RT-PCR

As previously described (5), TIANGEN reagent (TIANGEN Biotech, Beijing, China) was used to extract total RNA from homogenized tissue or cultured cells, following the manufacturer's instructions. The reaction solution consisted of 2.0 μl of diluted RT-PCR product, a 0.2 μm concentration of each paired primer, and power SYBR Green PCR master mix (Toyobo, Osaka, Japan). The Kv4.2 primer sequence was 5′-TGTCAGGAAGTCATAGAGGCAGCGTG-3′ (forward) and 5′-GGGGTGGTTACTGGAGGTGTTGGAAT-3′ (reverse). The sequence of housekeeping gene cyclophilin D, used as a control to exclude sampling errors, was 5′-GGACGTCTGTCTTCGAGTCC-3′ (forward) and 5′-AACAGACCGTGGAGATTTGG-3′ (reverse). The annealing temperature was set at 58 °C for Kv4.2 and 61 °C for cyclophilin D, with 38 amplification cycles for each product. The absolute mRNA levels in each sample were calculated according to a standard curve set up using serial dilutions of known amounts of specific templates against corresponding cycle threshold (*Ct*) values. The normalized ratio of Kv4.2 to cyclophilin D in each group was presented. The specificity of the primers was verified by both gel electrophoresis and sequencing of the PCR products.

##### Measurement of Intracellular Ca^2+^ Levels in CGNs

Intracellular Ca^2+^ levels were measured by using single cell fura-2 acetoxymethyl (AM) as described by Grynkiewicz *et al.* (51). Briefly, the CGNs were loaded with fura-2 AM (Invitrogen) and 0.02% pluronic F127 (Invitrogen) in Hanks' balanced salt solution (composition: 126.0 mm NaCl, 2.5 mm KCl, 2.0 mm MgSO_4_, 2.0 mm CaCl_2_, 10.0 mm d-glucose, and 10.0 mm HEPES, pH 7.4) at 37 °C for 45 min in darkness. CGNs were then rinsed three times in fura-2 AM-free Hanks' balanced salt solution at room temperature. The coverslip was mounted on an open slide chamber (containing 1 ml of fura-2 AM-free Hanks' balanced salt solution), and the chamber was put on an inverted Epi-fluorescence microscope (Nikon, Tokyo, Japan). The excitation wavelengths for fura-2 AM were 340/380 nm with emission at 505 nm. Baseline [Ca^2+^]*_i_* was determined for 60 s prior to the addition of K^+^ solution (27 mm KCl). The data were collected at 4-s intervals throughout the experiment. Quantification of the fluorescence intensity was performed using MetaFluor software (Universal Imaging Corporation, Milwaukee, WI).

Calibration of calcium imaging was done *in vitro* using a calcium calibration buffer kit (Invitrogen). The calibration results were plotted by double-log of the equation [Ca^2+^]_free_ = *K_d_* × [*R* − *R*_min_]/[*R*_max_ − *R*] × *F*_max_^380^/ *F*_min_^380^, where *R* indicates the ratio of 505-nm emission intensity with excitation at 340–505-nm emission intensity with excitation at 380 nm; *R*_min_ indicates the same ratio at zero-free Ca^2+^; *R*_max_ indicates the ratio at saturating Ca^2+^; *F*_max_^380^ is the fluorescence intensity with excitation at 380 nm for zero-free Ca^2+^; and *F*_min_^380^ is the fluorescence intensity at saturating free Ca^2+^. After linear fit, *K_d_* was acquired from the resulting straight line, and the Ca^2+^ concentration corresponding to *R* was calculated by the equation.

##### Immunocytochemistry

Cultured cells were fixed in fresh 4% paraformaldehyde in 0.1 m PBS for 15 min. Fixed cells were washed twice in ice-cold PBS and permeabilized with 0.25% Triton X-100 for 10 min. The cells were then washed three times in PBS for 5 min each and blocked in 1% BSA for 30 min. The labeling experiments were performed by incubating cells with antibody against NFATc4 (1:100, catalog no. 2183; Cell Signaling Technology, Danvers, MA) or actin (1:100, catalog no. AA128; Beyotime) at 4 °C overnight. After vigorous washing in PBS, the cells were incubated with the corresponding FITC-conjugated goat anti-rabbit IgG (1:200, catalog no. A0562; Beyotime) or Cy3-conjugated goat anti-mouse IgG (1:200, catalog no. A0521; Beyotime) for 1 h at room temperature. Fluorescence-labeled cells were visualized with a SP2 confocal laser scanning microscope (Leica, Mannheim, Germany) using a 40× objective lens.

##### Transfection and Luciferase Reporter Assays

Mouse Kv4.2 promoter (−2181 to +191 bp) and promoter Δ (−1513 to +191 bp) were subcloned into pGL3-Basic luciferase reporter plasmid. HeLa cells were co-transfected with Kv4.2 promoter and β-gal reporter plasmids. Luciferase activity prepared from transfected cell lysates was determined using a luciferase assay system (Promega, Madison, WI). Luciferase activity was presented as the ratio of luciferase to β-gal activity.

##### Chromatin Immunoprecipitation

ChIP was conducted according to the manufacturer's instructions (Beyotime). Sheared chromatin DNA was immunoprecipitated with the NFATc4 antibody or normal IgG. The antibody-protein-DNA complex was precipitated with protein A+G-agarose and collected for reverse cross-linking after washing. The DNA recovered was subjected to PCR amplification using primers as indicated.

##### Animals and AAV-mediated Injection

All experiments were conducted in strict accordance with the recommendations presented in the Guide for the Care and Use of Laboratory Animals (National Institutes of Health). The protocol was approved by the Committee on the Ethics of Animal Experiments of Fudan University (permit 20090614-001). Previously described *Nfatc2*^−/−^ and *Nfatc4*^−/−^ mice (52) were mated with C57BL/6 mice to obtain heterozygous *Nfatc2*^+/−^ *and Nfatc4*^+/−^ mice. After more than 10 generations of mating with C57BL/6 mice, back-crossed *Nfatc2*^+/−^ *and Nfatc4*^+/−^ mice were used to produce *Nfatc2*^−/−^ *and Nfatc4*^−/−^ mice. Three-week-old C57BL/6 or *Nfatc4*^−/−^ mice were injected with either AAV-control or AAV-neuritin virus (42). Virus particles (0.2 μl) were injected bilaterally into the PFC at a depth of 1 mm, 0.5–1 mm left and right of the brain raphe, and 2 mm before the bregma point. The mice were sacrificed 2 weeks after injection to prepare coronal brain slices (200 μm) containing PFC for Western blotting, patch clamp recording (47), or Golgi staining.

##### Dendritic Measurement

Identification of neurons was performed as described previously using Golgi staining (53). A homogenous neuronal population was selected for dendritic spine measurement based on the following criteria: (i) the cell body and dendrites were completely impregnated; (ii) the selected neurons were separated from the surrounding neurons; and (iii) all of the dendrites were visible within the plane of focus. Morphology and dendrite length of the selected cells were reconstructed using Neurolucida v9.0 software (MBF Bioscience, Williston, FL). Dendritic spines were counted on 20 μm of second order apical dendrites of pyramidal neurons in the PFC. Spine density was expressed as the average number of spines per micron of dendrite length. Sholl analysis was conducted to acquire branch intersections and lengths of the first order and second order apical dendrites (54).

##### Data Acquisition

The results were analyzed using Student's *t* test for comparison of two samples or between one sample and the hypothetical mean and one-way ANOVA with Fisher's post hoc test for comparisons between multiple groups. Cumulative frequency distributions of spine length were analyzed using a one-way Kruskal-Wallis test followed by the Kolmogorov-Smirnov test for post hoc comparisons. Intersection numbers were analyzed by two-way ANOVA with distance from soma and group as two factors. Fisher's post hoc test was used for mean comparison. The data were presented as individual data points using a scatter plot with *n* as the number of neurons for electrophysiological recordings, the number of imaging experiments, or the number of replicates for molecular biology experiments. For electrophysiological experiments, data were collected from at least four different batches of neurons prepared at different dates, thereby minimizing bias resulting from culture conditions. A *p* value of ≤0.05 was considered statistically significant.

## Author Contributions

J.-J. Y. and Q.-R. Z. designed, performed, and analyzed the experiments; D.-D. L. provided technical assistance and contributed to the preparation of the figures; and C.-W. C. and Y.-A. M. designed the study and wrote the paper. All authors reviewed the results and approved the final version of the manuscript.

## Footnotes

4 The abbreviations used are:

mTOR: mammalian target of rapamycin
IR: insulin receptor
CaN: calcineurin
CGN: cerebellum granule neuron
AP: action potential
NFAT: nuclear factor of activated T-cells
DKO: double knock-out
AAV: adeno-associated virus
ANOVA: analysis of variance
CMZ: calmidazolium chloride
CsA: cyclosporin A
HNMPA: hydroxy-2-naphthalenylmethyl phosphonic acid
PFC: prefrontal cortex
AM: acetoxymethyl.
